# Supplementary material for: Empirical methods for controlling false positives and estimating confidence in ChIP-Seq peaks
Source: BMC Bioinformatics. 2008 Dec 5;9:523. doi: 10.1186/1471-2105-9-523 (PMC2628906; doi:10.1186/1471-2105-9-523)
Supplement: Additional File 1 — A variety of html documents from the USeq web site detailing the available applications, their best usage, output file type descriptions, command line menus, etc. [file 1471-2105-9-523-S1.zip › USeqUserGuides/JavaDoc.zip!/JavaDoc/gata/main/AnnoSpecParams.html]

AnnoSpecParams


|  |  |  |
| --- | --- | --- |
| **Overview** \| **Package** \| **Class** \| **Use** \| **Tree** \| **Deprecated** \| **Index** \| **Help** | |  |
| PREV CLASS   **NEXT CLASS** | **FRAMES**    **NO FRAMES**     **All Classes** |  |
| SUMMARY: NESTED \| FIELD \| CONSTR \| METHOD | DETAIL: FIELD \| CONSTR \| METHOD |  |


---


## gata.main Class AnnoSpecParams

```
java.lang.Object
  gata.main.AnnoSpecParams
```

---

``` public class AnnoSpecParams extends java.lang.Object ```

**Author:**
:   Nix
    Contains fields specific for each annotation panel, either for the Reference Sequence or Comparative Sequence

---

**Constructor Summary**

`AnnoSpecParams()`


| **Method Summary** | |
| --- | --- |
| `void` | `addLabel(java.util.ArrayList list)` |
| `void` | `addNormalDNALines(java.util.ArrayList al)` |
| `void` | `addProteinLines(java.util.ArrayList thinLinesProt, java.util.ArrayList dottedLinesProt)` |
| `void` | `addRNALines(java.awt.geom.Line2D.Double baseLine, java.util.ArrayList fatLines)` |
| `void` | `addThinDNALines(java.util.ArrayList al)` |
| `void` | `clearLines()`             Wipes all the Line2D.Double ArrayLists, call before reDrawing the Glyphs when params have been changed. |
| `int` | `getAnnoPanelHeight()` |
| `Annotation` | `getAnnotation()` |
| `boolean` | `getAnyGenericGlyphs()` |
| `int` | `getBiggestGlyphPanelY()` |
| `java.util.ArrayList` | `getDottedProteinLines()` |
| `java.util.ArrayList` | `getFatRNALines()` |
| `GeneRepGlyph[]` | `getGeneGlyphs()` |
| `java.io.File` | `getGffFile()` |
| `GlyphPanel` | `getGlyphPanel()` |
| `java.awt.Graphics2D` | `getGraphics2DN()` |
| `java.util.ArrayList` | `getLabels()` |
| `java.util.ArrayList` | `getNormalDNALines()` |
| `double` | `getNtAtXZero()` |
| `double` | `getReferenceY()` |
| `ScaleBar` | `getScaleBar()` |
| `double` | `getScaleBarX()` |
| `double` | `getScaleBarY()` |
| `java.awt.Color` | `getScaleLineColor()` |
| `double` | `getScaleRulerY()` |
| `int` | `getSmallestGlyphPanelY()` |
| `java.util.ArrayList` | `getThinDNALines()` |
| `java.util.ArrayList` | `getThinProteinLines()` |
| `java.util.ArrayList` | `getThinRNALines()` |
| `GenericGlyph[][]` | `getTrackedGenericGlyphs()` |
| `java.util.ArrayList[]` | `getTrackLabels()` |
| `boolean[]` | `getTrackLabelVis()` |
| `int` | `getTrackNumber()` |
| `java.awt.BasicStroke[]` | `getTrackStrokes()` |
| `float[]` | `getTrackThickness()` |
| `boolean[]` | `getTrackVis()` |
| `boolean` | `isScaleBarVis()` |
| `boolean` | `isScaleRulerVis()` |
| `void` | `setAnnoPanelHeight(int i)` |
| `void` | `setAnnotation(Annotation annotation)` |
| `void` | `setAnyGenericGlyphs(boolean x)` |
| `void` | `setBiggestGlyphPanelY(int i)` |
| `void` | `setGeneGlyphs(GeneRepGlyph[] glyphs)` |
| `void` | `setGffFile(java.io.File file)` |
| `void` | `setGlyphPanel(GlyphPanel panel)` |
| `void` | `setGraphics2DN(java.awt.Graphics2D g2)` |
| `void` | `setNtAtXZero(double d)` |
| `void` | `setReferenceY(double d)` |
| `void` | `setScaleBar(ScaleBar bar)` |
| `void` | `setScaleBarVis(boolean b)` |
| `void` | `setScaleBarX(double d)` |
| `void` | `setScaleBarY(double d)` |
| `void` | `setScaleLineColor(java.awt.Color color)` |
| `void` | `setScaleRulerVis(boolean b)` |
| `void` | `setScaleRulerY(double d)` |
| `void` | `setSmallestGlyphPanelY(int i)` |
| `void` | `setTrackedGenericGlyphs(GenericGlyph[][] glyphs)` |
| `void` | `setTrackLabels(java.util.ArrayList[] lists)` |
| `void` | `setTrackLabelVis(boolean[] bs)` |
| `void` | `setTrackNumber(int i)` |
| `void` | `setTrackThickness(float[] fs)` |
| `void` | `setTrackVis(boolean[] bs)` |

**Methods inherited from class java.lang.Object**

`equals, getClass, hashCode, notify, notifyAll, toString, wait, wait, wait`

**Constructor Detail**

### AnnoSpecParams

```
public AnnoSpecParams()
```


**Method Detail**

### addLabel

```
public void addLabel(java.util.ArrayList list)
```

---


### addNormalDNALines

```
public void addNormalDNALines(java.util.ArrayList al)
```

---


### addProteinLines

```
public void addProteinLines(java.util.ArrayList thinLinesProt,
                            java.util.ArrayList dottedLinesProt)
```

---


### addRNALines

```
public void addRNALines(java.awt.geom.Line2D.Double baseLine,
                        java.util.ArrayList fatLines)
```

---


### addThinDNALines

```
public void addThinDNALines(java.util.ArrayList al)
```

---


### clearLines

```
public void clearLines()
```

:   Wipes all the Line2D.Double ArrayLists, call before reDrawing
    the Glyphs when params have been changed.

---


### getAnnoPanelHeight

```
public int getAnnoPanelHeight()
```

---


### getAnnotation

```
public Annotation getAnnotation()
```

---


### getAnyGenericGlyphs

```
public boolean getAnyGenericGlyphs()
```

---


### getDottedProteinLines

```
public java.util.ArrayList getDottedProteinLines()
```

---


### getFatRNALines

```
public java.util.ArrayList getFatRNALines()
```

---


### getGeneGlyphs

```
public GeneRepGlyph[] getGeneGlyphs()
```

---


### getGlyphPanel

```
public GlyphPanel getGlyphPanel()
```

---


### getGraphics2DN

```
public java.awt.Graphics2D getGraphics2DN()
```

---


### getLabels

```
public java.util.ArrayList getLabels()
```

---


### getNormalDNALines

```
public java.util.ArrayList getNormalDNALines()
```

---


### getReferenceY

```
public double getReferenceY()
```

---


### getThinDNALines

```
public java.util.ArrayList getThinDNALines()
```

---


### getThinProteinLines

```
public java.util.ArrayList getThinProteinLines()
```

---


### getThinRNALines

```
public java.util.ArrayList getThinRNALines()
```

---


### getTrackLabelVis

```
public boolean[] getTrackLabelVis()
```

---


### getTrackVis

```
public boolean[] getTrackVis()
```

---


### getTrackedGenericGlyphs

```
public GenericGlyph[][] getTrackedGenericGlyphs()
```

---


### getTrackLabels

```
public java.util.ArrayList[] getTrackLabels()
```

---


### getTrackNumber

```
public int getTrackNumber()
```

---


### setAnnotation

```
public void setAnnotation(Annotation annotation)
```

---


### setAnyGenericGlyphs

```
public void setAnyGenericGlyphs(boolean x)
```

---


### setGeneGlyphs

```
public void setGeneGlyphs(GeneRepGlyph[] glyphs)
```

---


### setGlyphPanel

```
public void setGlyphPanel(GlyphPanel panel)
```

---


### setGraphics2DN

```
public void setGraphics2DN(java.awt.Graphics2D g2)
```

---


### setTrackLabelVis

```
public void setTrackLabelVis(boolean[] bs)
```

---


### setTrackVis

```
public void setTrackVis(boolean[] bs)
```

---


### setTrackedGenericGlyphs

```
public void setTrackedGenericGlyphs(GenericGlyph[][] glyphs)
```

---


### setTrackLabels

```
public void setTrackLabels(java.util.ArrayList[] lists)
```

---


### setTrackNumber

```
public void setTrackNumber(int i)
```

---


### setAnnoPanelHeight

```
public void setAnnoPanelHeight(int i)
```

---


### getSmallestGlyphPanelY

```
public int getSmallestGlyphPanelY()
```

---


### setSmallestGlyphPanelY

```
public void setSmallestGlyphPanelY(int i)
```

---


### getBiggestGlyphPanelY

```
public int getBiggestGlyphPanelY()
```

---


### setBiggestGlyphPanelY

```
public void setBiggestGlyphPanelY(int i)
```

---


### setReferenceY

```
public void setReferenceY(double d)
```

---


### getScaleRulerY

```
public double getScaleRulerY()
```

---


### setScaleRulerY

```
public void setScaleRulerY(double d)
```

---


### getScaleBar

```
public ScaleBar getScaleBar()
```

---


### setScaleBar

```
public void setScaleBar(ScaleBar bar)
```

---


### getNtAtXZero

```
public double getNtAtXZero()
```

---


### setNtAtXZero

```
public void setNtAtXZero(double d)
```

---


### getGffFile

```
public java.io.File getGffFile()
```

---


### setGffFile

```
public void setGffFile(java.io.File file)
```

---


### getTrackStrokes

```
public java.awt.BasicStroke[] getTrackStrokes()
```

---


### setTrackThickness

```
public void setTrackThickness(float[] fs)
```

---


### getTrackThickness

```
public float[] getTrackThickness()
```

---


### getScaleLineColor

```
public java.awt.Color getScaleLineColor()
```

---


### setScaleLineColor

```
public void setScaleLineColor(java.awt.Color color)
```

---


### isScaleBarVis

```
public boolean isScaleBarVis()
```

---


### isScaleRulerVis

```
public boolean isScaleRulerVis()
```

---


### setScaleBarVis

```
public void setScaleBarVis(boolean b)
```

---


### setScaleRulerVis

```
public void setScaleRulerVis(boolean b)
```

---


### getScaleBarX

```
public double getScaleBarX()
```

---


### getScaleBarY

```
public double getScaleBarY()
```

---


### setScaleBarX

```
public void setScaleBarX(double d)
```

---


### setScaleBarY

```
public void setScaleBarY(double d)
```


---


|  |  |  |
| --- | --- | --- |
| **Overview** \| **Package** \| **Class** \| **Use** \| **Tree** \| **Deprecated** \| **Index** \| **Help** | |  |
| PREV CLASS   **NEXT CLASS** | **FRAMES**    **NO FRAMES**     **All Classes** |  |
| SUMMARY: NESTED \| FIELD \| CONSTR \| METHOD | DETAIL: FIELD \| CONSTR \| METHOD |  |


---
